# Description of the species of *Dicoelothorax* Ashmead (Chalcidoidea, Eucharitidae) and biology of *D. platycerus* Ashmead

**DOI:** 10.3897/zookeys.165.2089

**Published:** 2012-01-13

**Authors:** Javier Torréns, John M. Heraty

**Affiliations:** 1 CRILAR-CONICET, Entre Ríos y Mendoza, 5301 Anillaco, La Rioja, Argentina; 2 Department of Entomology, University of California, Riverside, CA, USA 92521

**Keywords:** *Dicoelothorax*, eggs, planidia, pupae, host ant, host plant

## Abstract

Descriptions of the adults of the two species of *Dicoelothorax* Ashmead, *Dicoelothorax parviceps* and *Dicoelothorax platycerus*, and the eggs, planidia and pupae of *Dicoelothorax platycerus* Ashmead are provided. Females of *Dicoelothorax platycerus* deposit their eggs on the underside of leaves of *Pseudabutilon virgatum* (Cav.) Fryxell (Malvaceae). The host of *Dicoelothorax platycerus* is *Ectatomma brunneum* Smith (Formicidae: Ectatomminae).

## Introduction

Eucharitidae are parasitoids of pupae of Formicidae (Hymenoptera: Aculeata) (Clausen 1940, Heraty 2002), females lay their eggs away from the host within the tissues of certain plants and the active first-instar larva, termed a planidium, must make its way back to the ant nest where it can attack the brood (Heraty and Barber 1990).

*Dicoelothorax* was established by Ashmead (1899), but without a type species. Ashmead (1904) later designated *Dicoelothorax platycerus* as the type species. Heraty (2002) provides a detailed diagnosis and description of the genus, and a morphological phylogenetic analysis placing this genus within a Neotropical *Kapala* clade that are all parasitoids of poneromorph ants. A sister group relationship with *Lasiokapala* Ashmead, 1899 was proposed based on the broad, angulate postgenal margin and the absence of a postmarginal vein (Heraty 2002).

This genus includes two species distributed in the Neotropical region: *Dicoelothorax parviceps* Cameron (Argentina, Brazil, Colombia and Guyana) and *Dicoelothorax platycerus* Ashmead (Argentina, Bolivia and Brazil). The original descriptions of these species are vague and short, and there is no clear differentiation of species. Based on the collections examined and our new material, we were able to differentiate both species. Herein we provide new descriptions and diagnoses. Also, *Dicoelothorax platycerus* was collected in northwestern Argentina, and information on life history, immature stages, and a new host association are included.

## Materials and methods

*Dicoelothorax platycerus* were collected at San Vicente, Tucumán (26°25'36"S, 65°15'41"W; 740 m altitude) on March 12, 2009 on *Pseudabutilon virgatum* (Cav.) Fryxell (Malvaceae). Eggs were found on the underside of the leaves. Five females of *Dicoelothorax platycerus* were collected in the field and provided twigs with leaves, fruits, and flowers of different species of plants in 10 × 3.5 cm plastic tubes to monitor oviposition habits. Leaves of *Pseudabutilon virgatum* with eggs were placed into a cylindrical glass container of 10 × 10 cm with dampened cotton until emergence of the first instar (planidium). The planidia and some eggs were preserved in ethanol. Planidia were cleared in 10% KOH and both larvae and eggs slide-mounted in Hoyer’s medium.

Three nests of *Ectatomma brunneum* Smith (Formicidae: Ectatomminae) that were in close proximity to the adult collection and oviposition site were excavated. Adults, brood, and debris were collected into plastic containers. Adults and immature stages were then sorted from the debris, examined for parasitism, and subsequently returned to the containers to allow further development of immatures. The immature stages were examined once daily until all parasitoids or ants emerged from the cocoons.

Images were obtained using GT-VISION® ENTO-VISION software operating on a Leica M16 zoom lens linked to a JVC KY-F75U 3-CCD digital video camera; and LEICA APPLICATION SUIT (version 3.5.0) software operating on a Leica MZ12 linked to a Leica DFC295 digital video camera. Images were enhanced with COREL PHOTOPAINT and COREL DRAW (version 15); and some images processed with DEEP FOCUS (Stuart Ball).

Specimens studied are deposited in the Museo Argentino de Ciencias Naturales “Bernardino Rivadavia”, Buenos Aires, Argentina (MACN); Instituto Fundación Miguel Lillo, Tucumán, Argentina (IFML); University of California, Riverside, California, USA (UCRC); and American Museum of Natural History, New York, USA (AMNH). Notes and detailed illustrations of the type material housed at the National Museum of Natural History, Washington (USNM) and the Natural History Museum, London (BMNH) were made available by J. Heraty (UCRC).

Morphological terms are from Heraty (2002) and Heraty and Darling (1984), with details on sculpture from Eady (1968) and Harris (1979).

## Taxonomy

### Dicoelothorax parviceps

Cameron

http://species-id.net/wiki/Dicoelothorax_parviceps

Figs 1–5

Dicoelothorax parviceps Cameron 1913: 117–118; De Santis 1979: 107; De Santis 1980: 211; Heraty 2002: 130. Type female in BMNH 5.364 [examined], UCRC_ENT 310015.

#### Description.

 Distinguished from *Dicoelothorax platycerus* by the mesosoma and frenal processes having distinct, widely spaced longitudinal striae (Figs 2, 5); dorsal concavity of mesoscutum and scutellum smooth medially (Fig. 2); frenal processes in dorsal view distinctly tapered with apex narrowly rounded (Figs 2, 5); venation yellow with stigma pale brown; scutellar processes of male white and straight in lateral view, and 1.6× as long as scutellum (Figs 4, 5).

**Female.** Length3.8 mm. Head, mesosoma, coxae, petiole and Gt_1_ except distal part black; basal ¾ of femora, frenal processes, distal part of Gt_1_ and rest of terga brown; antenna yellowish to light brown; rest of legs and distal limits of terga yellowish. Wings slightly infuscate, venation yellow, stigmal vein pale brown (Fig. 3).

**Figures 1–5.**
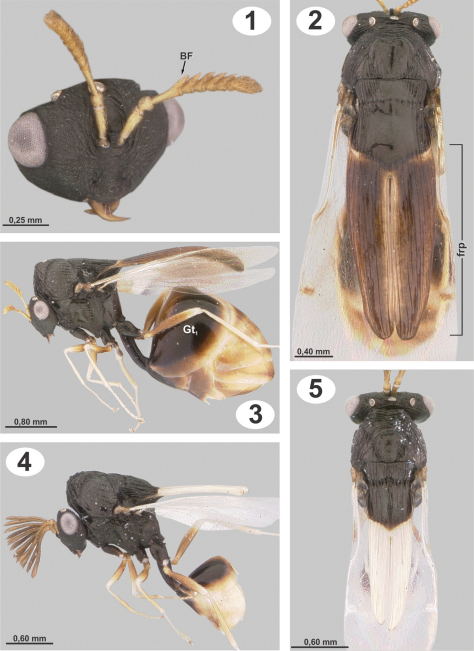
*Dicoelothorax parviceps* **1** head and antenna (female, sublateral) **2** mesosoma (female, dorsal) **3** habitus (female) **4** habitus (male) **5** mesosoma (male, dorsal).

*Head* 1.7× as broad as high. Frons and face granulate, weakly strigose, with small and scattered setae (Fig. 1). Eyes separated by 2.3× their height. Malar space as long as height of eye. Antenna with 8 segments; scape 2.9× as long as broad, broader apically, smooth, with a few scattered setae. Length of flagellum 0.9× height of head, basal flagellomere (homologous to F1+F2; Heraty 2002) as long as scape (BF, Fig. 1), following flagellomeres serrate, clava rounded (Fig. 1).

*Mesosoma.* Midlobe of mesoscutum elevated anteriorly, with short, thin, decumbent and scattered setae; striate-rugose on anterior face of mesoscutum, sidelobes longitudinally striate, midlobe dorsally smooth and concave (Figs 2, 3). Axilla and scutellar disc smooth and concave dorsally, scutellar disc longitudinally striate laterally. Scutoscutellar sulcus (SSS) weakly crenulate dorsally; deeply invaginated and smooth laterally. In dorsal view, frenal processes tapering toward narrowly rounded apex, with longitudinal striae distinct and widely spaced; processes 3.8× as long as maximum width and 2.1× as long as scutellum (frp, Fig. 2), in profile curved over gaster. Upper half of mesepisternum and mesepimeron longitudinally striate. Hind coxa semiglobose, 1.9× as long as broad; with weak longitudinal striae and scattered, thin setae. Hind femur densely setose. Forewing 2.8× as long as broad; stigmal vein slender and perpendicular to wing margin, 3.3× as long as broad; postmarginal vein indistinct but present and about as long as stigmal vein (Fig. 2).

*Metasoma*. Petiole 4.1× as long as broad, 1.8× as long as hind coxa and 1.2× as long as hind femur; First gastral tergite (Gt_1_) smooth and without setae (Gt_1_, Fig. 3).

**Male.** Length 2.4 mm. Similar to female except for following. Antenna brown, frenal processes and venation completely white; Gt_1_ and following segments yellowish (Figs 4, 5); forewing hyaline. Head 1.3× as long as high. Eyes separated by 2.1× their height. Malar space 0.9× as height of eyes. Antenna pectinate; scape shorter than female, 2.4× as long as broad; branch of basal flagellomere 0.9× as long as height of head, following flagellomeres with branches progressively decreasing in length (Fig. 4). Mesoscutal depression rugose (Fig. 5); axilla and scutellar disc narrower than mesoscutum and with longitudinal striae. SSS deeply crenulate dorsally. Frenal processes smaller than female; frenal processes 4.3× as long as maximum width, 1.6× as long as scutellum; spines straight in lateral view (Figs 4, 5). Mesepisternum and mesepimeron with weak striae. Hind coxa 1.8× as long as broad. Petiole 3.4× as long as broad, 2.0× as long as hind coxa.

#### Biology of Dicoelothorax parviceps.

 Unknown.

#### Material examined.

 COLOMBIA. Vichada, P. N. Tuparro, 16.vi.2000, Sharkey, UCRC_ENT 161564 (1 female, UCRC); same location and data, UCRC_ENT 92180 (1 male, UCRC).

### Dicoelothorax platycerus

Ashmead

http://species-id.net/wiki/Dicoelothorax_platycerus

**Figures 6–10.**
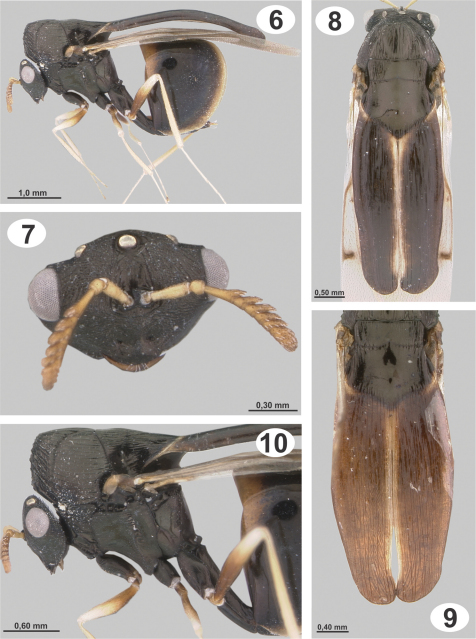
*Dicoelothorax platycerus*(female) **6** habitus **7** head and antenna (sublateral) **8** mesosoma (dorsal) **9** scutellum (dorsal) **10** mesosoma (lateral).

Figs 6 [Fig F3] [Fig F4] [Fig F5] –31

Dicoelothorax platycerus Ashmead 1904: 470–471; De Santis 1979: 107; De Santis 1980: 211; Heraty 2002: 130, figs 113–119 (lectotype and paralectotype). Type females in USNM, http://www.chalcidtypes.com/default.asp?Action=Show_Types&Single_Type=True&TypeID=878 [examined]

#### Description.

Distinguished from *Dicoelothorax parviceps* by the mesosoma and frenal processes having fine closely-spaced longitudinal striae, closer and more slightly raised in female (Figs 8, 9, 12, 14); dorsal concavity of mesoscutum and scutellum smooth or weakly striate medially (Figs 8, 9); frenal processes in dorsal view widened medially and narrowing only slightly to apex, which is almost the same width as their base and broadly rounded (Figs 8, 9); venation brown; scutellar processes of male yellowish with diffuse black longitudinal band medially and apex black, slightly curved in lateral view, and almost twice as long as scutellum (Figs 12, 14).

**Female.** Length 3.0–4.5 mm. Head, mesosoma, coxae, petiole and Gt_1_ except distal part black; flagellum, basal ¾ of femora, frenal process, distal part of Gt_1_ and rest of terga brown but with processes sometimes completely black; scape, pedicel and rest of legs and distal limits of terga yellowish (Figs 6, 8, 9). Wings slightly infuscate, venation brown.

*Head* 1.4–1.5× as broad as high. Frons and face granulate, weakly strigose, with small and scattered setae or without setae (Fig. 7). Eyes separated by 2.3–2.7× their height. Malar space 0.8–1.2× as long as height of eyes. Antenna with 8 segments; scape 2.4–2.8× as long as broad, slightly broader apically, smooth, with a few scattered setae. Length of flagellum 0.7–0.9× height of head, basal flagellomere 0.8–1.2× as long as scape, basal flagellomere ranging from serrate to clavate, following flagellomeres serrate, clava rounded (Fig. 7).

*Mesosoma.* Midlobe of mesoscutum elevated anteriorly, with short, thin, decumbent and scattered setae; striate-rugose on anterior face, sidelobes longitudinally striate, modlobe dorsally smooth or weakly striate and concave (Figs 6, 10). Axilla and scutellar disc smooth and concave dorsally, scutellar disc longitudinally striate laterally. SSS weakly crenulate dorsally and deeply invaginated and smooth laterally (Figs 8, 9). In dorsal view, frenal processes widened medially and tapering only slightly to apex, apically almost the same width as their base and broadly rounded, with longitudinal striae slightly marked and closely spaced; processes 3.1–3.5× as long as maximum width and 2.4–2.7× as long as scutellum (Figs 6, 8, 9); in profile, curved over gaster. Upper half of mesepisternum and mesepimeron longitudinally striate. Hind coxa semiglobose and elongate, 1.7–2.1× as long as broad; with weak longitudinal striae and scattered, thin setae (Fig. 10). Hind femur densely setose. Forewing 2.3–2.5× as long as broad; stigmal vein slender and perpendicular to wing margin, 1.9–2.2× as long as broad; postmarginal vein indistinct and less than half as long as stigmal vein (Fig. 8).

*Metasoma*. Petiole 3.6–4.1× as long as broad, 1.7–2.0× as long as hind coxa and 1.2–1.3× as long as hind femur; Gt_1_ smooth and without setae (Figs 6, 10).

**Figures 11–14. F3:**
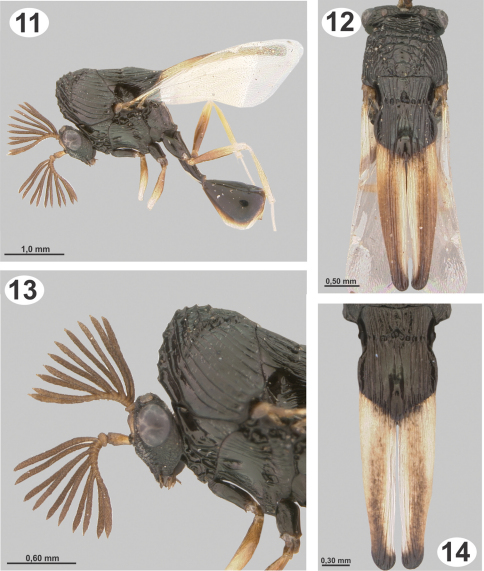
*Dicoelothorax platycerus* (male) **11** habitus **12** mesosoma (dorsal) **13** head and mesoscutum (lateral) **14** scutellum (dorsal).

**Male.** Length 3.0–3.8 mm. Similar to female except for following. Antenna brown, frenal processes yellowish with a diffuse black longitudinal band medially and apex black, this band can be extended laterally and covering almost entire surface, or it can be reduced to a narrow medial line (Figs 11, 12, 14); wing venation white, forewing hyaline. Head 1.5–1.6× as long as high. Eyes separated by 2.2–2.4× their height. Malar space 0.7–0.9× as height of eyes. Antenna pectinate; scape shorter than female, 1.8–1.9× as long as broad; basal flagellomere 0.9–1.0× as long as height of head, following flagellomeres with branches progressively decreasing in length (Fig. 13). Mesosoma with striae stronger than female, mesoscutal depression rugose (Fig. 12); axilla and scutellar disc narrower than mesoscutum and with longitudinal striae; scutellum with a small depression anterior to union of processes (Figs 12, 14). SSS deeply crenulate dorsally. Frenal processes narrowing toward apex; 3.7–4.4× as long as maximum width, 1.7–2.1× as long as scutellum (Figs 12, 14); in profile, uniformly and slightly curved over gaster. Hind coxa 1.8–2.1× as long as broad. Petiole 3.8–4.3× as long as broad, 1.7–2.1× as long as hind coxa. Gaster smaller than female.

**Eggs.** Length of egg body 0.18 mm and caudal stalk 0.08 mm (Fig. 19). Undeveloped eggs are whitish and translucent with a smooth chorion, slightly flattened dorsally and convex ventrally, with a caudal stalk that is about half the length of the egg body. The egg is similar to other Eucharitinae as described by Heraty and Darling (1984).

**Figures 15–20. F4:**
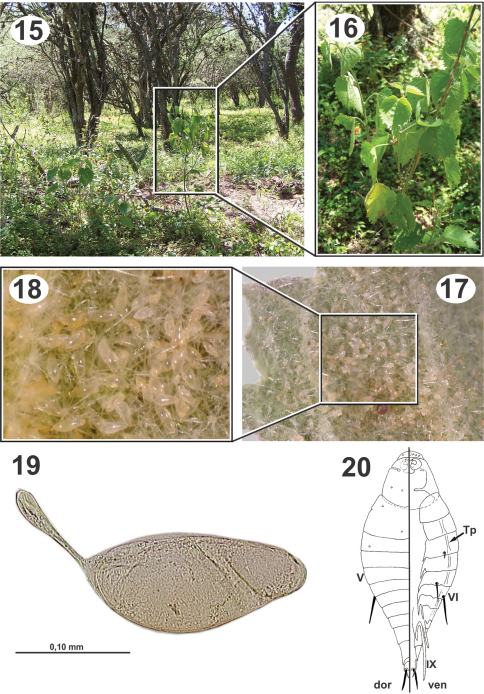
Biology and immature stages of *Dicoelothorax platycerus* **15** habitat **16** *Pseudabutilon virgatum* **17** underside leaf of *Pseudabutilon virgatum* with eggs **18** magnified area with eggs **19** egg **20** planidium.

#### Planidium.

As described for other Eucharitinae by Heraty and Darling (1984), but distinguished as follows: length 0.09 mm, width 0.05 mm (Fig. 20); pleurostomal spine not observed; anterior pair of placoid sensilla connected to lateral margin by single line of weakness, dorsal cranial spines absent; ventral transverse process of cranium fingerlike; tergopleural line (Tp) separating pleural and dorsal tergites present on tergites TII–VIII; TI and TII fused dorsally, with two pair of small setae dorsally; TIII with one pair of setae ventrally and one pair dorsally; TV with one pair of stout setae ventrally, reaching to TVII; TVI with one pair of stout setae lateral to Tp; TIX entire and with two long lateral processes ventrally reaching to middle of caudal cerci; TXII with lateral processes reaching to almost the middle of caudal cerci; caudal cerci stout (Fig. 20).

#### Pupa.

 Length: 5.4–6.7 mm (Figs 26–31). The pupa are similar to the description by Pérez-Lachaud et al. (2006a) for *Kapala izapa* Carmichael, but differ as follows: with blunt conical projections on each sidelobe of mesoscutum (Figs 27, 29); one pair of conical and pointed projections in the axilla; undeveloped frenal processes broad and flattened; gaster with raised ridges along metasomal tergites, the first tergite with lateral and ventral projections, and following segments with dorsal, lateral and ventral projections. The larval exuvium was attached to the terminal segments of the gaster (Figs 28, 30, 31). Pupation occurs inside of the ant cocoon (Fig. 26).

**Figures 21–25. F5:**
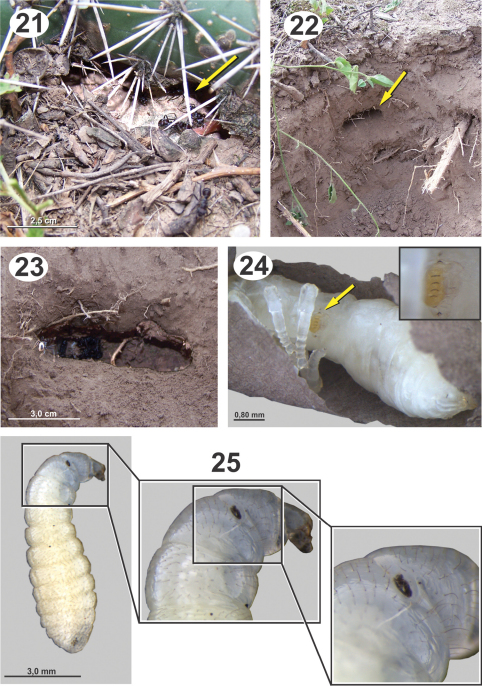
Biology and immature stages of *Dicoelothorax platycerus* **21** nest entrace of *Ectatomma brunneum* (opening indicated) **22** brood chamber (indicated) **23** brood chamber magnified **24** prepupa parasitized (2nd instar larva indicated and magnified) **25** ant larva parasitized (attached planidium magnified).

**Figures 26–31.**
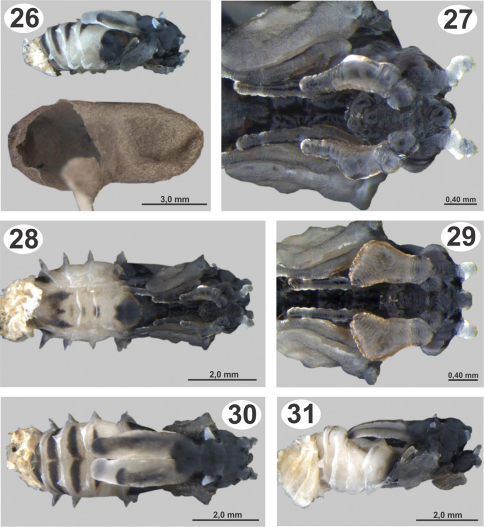
Pupae of *Dicoelothorax platycerus* **26** pupa extracted with ant cocoon (female, lateral) **27** head (female, ventral) **28** pupa in ventral view (female) **29** head (male, ventral) **30** pupa in dorsal view (female) **31** pupa in lateral view (male).

#### Habitat and location.

 Specimens were collected in San Vicente (Tucumán, Argentina). In this region it is common to find *Aspidosperma quebracho-blanco* Schlecht.(Quebracho blanco), *Cassia*, *Cercidium* sp. (Brea), *Cereus validus* Haworth, *Harrisia pomanensis* (F.A.C.Weber) Britton & Rose, *Jodina rhombifolia* Hooker et Arnott (Sombra de toro), *Opuntia* sp. (Tuna, Quimilo), and *Prosopis* sp. (Algarrobo). This vegetation corresponds to the chaco serrano ecoregion (*sensu* Digilio and Legname 1966). The host plant, *Pseudabutilon virgatum*, was widely distributed, but the specimens associated with *Dicoelothorax* were collected in a forest of *Prosopis* sp., 12 meters north of the road (Fig. 15).

#### Host Plant.

*Pseudabutilon virgatum* is a ligneous shrub that grows not more than 1 m in height, persists year round, and blooms in the humid seasons (spring-summer); its leaves are ovate and marginally serrate and last to the beginning of the cold season (May-June) (Fig. 16).

#### Host ants.

*Ectatomma brunneum* workers were observed and sampled from under the plants with *Dicoelothorax*. In a radius of about 4m, we found three ant nests (H1–H3). The disposition of chambers and general structure of nests are similiar to those observed by Lapola et al. (2003),. Nests had 1 to 3 openings at ground level, without any structure elevated above the surface (Fig. 21). Chambers from which the immature stages were extracted were found at a depth of 10 to 13 cm (Figs 22, 23). In two of those nests we found immature stages of ants and parasitoids; in the other (H3) we only found a chamber with a collection of arthropods suggesting that it was a food cache. Nest H1 contained 17 cocoons and 2 larvae, and nest H2 had 97 larvae and no cocoons.

#### Life History of Dicoelothorax platycerus.

 Collections of adults of *Dicoelothorax platycerus*, *Pseudabutilon virgatum*, and ant nests were made in 2009 (March 12) and 2010 (March 27 and April 3). Females placed in plastic tubes were observed ovipositing on the undersides of the leaves of *Pseudabutilon virgatum* (Figs 17, 18). A single gravid female oviposited about 40 eggs per 1 mm^2^ between the spicules forming the pubescence on the underside of leaves (Figs 17, 18). Numerous mites were observed on the leaves, and oviposition under the dense network of spicules appears to be a protection against egg predators. Eggs hatched within 10 days; however, many of the remaining eggs contained mature planidia that did not hatch. First instars (planidia) are very mobile and have a propensity to jump. Larvae presumably attach phoretically to foraging ants under the host plant and get carried back to the ant nest where they attack the ant larvae (Clausen 1941). Of two pupae of *Dicoelothorax platycerus* obtained in H1, one male emerged 12 days after the nest was excavated; whereas the other pupa (female) did not emerge (Figs 26–31). The percentage of parasitism ranged from 6.2% in H2 to 21% in H1. In nest H1, 17 cocoons were recovered, with two pupae of *Dicoelothorax platycerus* (1 female and 1 male) and 2 ant prepupae parasitized by second instars of *Dicoelothorax platycerus* (Fig. 24). In nest H2, 97 larvae were recovered with 6 parasitized by planidia (Fig. 25).

#### Discussion.

*Ectatomma brunneum* was reported as the ant host for an unidentified species of *Kapala* (Eucharitidae: Eucharitini) in French Guiana, (Lachaud et al. 2011). It is noteworthy that the same ant species is the primary host for at least two different eucharitid genera. Similarly, *Ectatomma tuberculatum* (Olivier) can be attacked by three different eucharitid genera, *Dilocantha*, *Isomerala* and *Kapala* (Pérez-Lachaud et al. 2006b).

#### Material examined.

ARGENTINA. Salta, Tartagal, xii.1971, UCRC_ENT 305490 and UCRC_ENT 305491 (2 males, AMNH). Salta, Güemes, 7.ii.1983, UCRC_ENT 305492 (1 female, AMNH); same location and data, UCRC_ENT 305493 (1 male, AMNH). Salta, Cabeza de Buey, 24°47'36"S, 64°01'57"W, 15–16.iii.2007, J.&J. Heraty & J. Torréns, UCRC_ENT 305494 (1 female, UCRC); same location and data, UCRC_ENT 305495 and UCRC_ENT 305496 (2 males, UCRC); same location and data, UCRC_ENT 305497, UCRC_ENT 305498 and UCRC_ENT 305499 (3 males, IFML). Salta, Cabeza de Buey, 24°47'36"S, 64°01'57"W, 19.ii.2008, P. Fidalgo, UCRC_ENT 305500 (1 female, MACN); same location and data, UCRC_ENT 305501 (1 male, MACN). Salta, Lumbreras, 25°12'19"S, 64°54'34"W, 14.iii.2009, J. Torréns, UCRC_ENT 305502 (1 female, IFML). Tucumán, San Vicente, 26°25'36"S, 65°15'41"W, 12.iii.2009, J. Torréns, UCRC_ENT 305503 and UCRC_ENT 305504 (2 females, IFML); same location, 27.iii.2010, J. Torréns, ex. Pupa of *Ectatomma* brunneum UCRC_ENT 305505 (1 female, IFML); same location and data, UCRC_ENT 305506 (1 male, IFML); same location, 03.iv.2010, J. Torréns, ex. pupa of *Ectatomma brunneum*, UCRC_ENT 305507 (1 male, IFML).

## Supplementary Material

XML Treatment for Dicoelothorax parviceps

XML Treatment for Dicoelothorax platycerus
